# Supplementary material for: Cardioprotective Effects of a Nonsteroidal Mineralocorticoid Receptor Blocker, Esaxerenone, in Dahl Salt-Sensitive Hypertensive Rats
Source: Int J Mol Sci. 2021 Feb 19;22(4):2069. doi: 10.3390/ijms22042069 (PMC7922950; doi:10.3390/ijms22042069)
Supplement: Supplementary file 1 [file ijms-22-02069-s001.pdf]

Supplementary Table 1. List of primers

Gene name	Primer sequences (5′–3′)	Product size
NPPA	F: CCTGGACTGGGGAAGTCAAC<br>R: ATCTATCGGAGGGGTCCCAG	78
NPPB	F: CAGAAGCTGCTGGAGCTGATA<br>R: GGCGCTGTCTTGAGACCTAA	120
TGF-β1	F: ACGGAAGCGCATCGAAGCCA<br>R: ACAAAGCGAGCACCGCCTCG	116
Collagen I	F: ACCTCCCGCCTGCCCATCAT<br>R: CACGAAGCAGGCAGGGCCAA	93
Collagen III	F: CCATGGGTCCCAGAGGGGCT<br>R: GGGACCTGGTTGCCCGTCAC	110
PAI-1	F: TCTCCGCCATCACCAACATT<br>R: GAGAGAACTTAGGCAGGATGAGG	99
TNF-α	F: CATCCGTTCTCTACCCAGCC<br>R: AATTCTGAGCCCGGAGTTGG	146
IL-6	F: TCTGTCTCGAGCCCACCAGGAA<br>R: CTGGCTGGAAGTCTCTTGCGGA	91
CXCL8	F: CCCCCATGGTTCAGAAGATTG<br>R: TTGTCAGAAGCCAGCGTTCAC	113
$gp47^{phox}$	F: GGATCACAGAAGGTCCCTAGC<br>R: AGAAGTTCAGGGCGTTCACC	139
$p22^{phox}$	F: TGGCCTGATCCTCATCACAG<br>R: AGGCACGGACAGCAGTAAGT	99
SGK-1	F: CGAGTCCGTCCTGCTAAGCG<br>R: TAGGATTGAACTTCAGGGTGTTTGC	120
18s	F: TTGATTAAGTCCCTGCCCTTTGT<br>R: CGATCCGAGGGCCTCACTA	77
